# HbA1c as a Continuous Marker of Microvascular Vulnerability: Development of a Non-Linear Risk Framework in a Real-World Cohort

**DOI:** 10.3390/metabo16030197

**Published:** 2026-03-16

**Authors:** Mihaela Simona Popoviciu, Alina Manuela Pop, Timea Claudia Ghitea, Florica Ramona Dorobantu, Carmen Pantis, Nicolae Ovidiu Pop, Roxana Daniela Brata

**Affiliations:** 1 Department of Preclinical Disciplines, Faculty of Medicine and Pharmacy, University of Oradea, 410028 Oradea, Romania; mihaela.popoviciu@didactic.uoradea.ro (M.S.P.); alinatirb@uoradea.ro (A.M.P.); 2 Department of Internal Medicine II, Diabetes Mellitus, Clinical County Emergency Hospital of Oradea, 410167 Oradea, Romania; 3 Pharmacy Department, Faculty of Medicine and Pharmacy, University of Oradea, 1 University Street, 410087 Oradea, Romania; 4 Department of Medical Disciplines, Faculty of Medicine and Pharmacy, University of Oradea, 410028 Oradea, Romania; rdorobantu@uoradea.ro (F.R.D.); pop.nicolaeovidiu@didactic.uoradea.ro (N.O.P.); brata.roxanadaniela@didactic.uoradea.ro (R.D.B.)

**Keywords:** HbA1c, continuous risk, metabolic stress, prediabetes, gray zone, non-linear modeling, spline analysis, microvascular complications, risk estimation

## Abstract

Background: Glycated hemoglobin (HbA1c) is widely used for the diagnosis and monitoring of diabetes mellitus; however, its interpretation is largely based on fixed diagnostic thresholds. This study moves beyond describing a glycemic continuum by translating the non-linear HbA1c–microvascular relationship into an individualized risk estimation framework. Methods: In this cross-sectional observational study, adult subjects from a real-world clinical cohort were analyzed using HbA1c as a continuous variable. Associations between HbA1c and metabolic parameters were assessed using correlation analysis. Linear regression was applied to evaluate the relationship between HbA1c and cumulative diabetes-related complication burden. Non-linear associations between HbA1c and the risk of presenting at least one complication were explored using restricted cubic spline logistic regression models. Additional risk estimation analyses focused on the HbA1c gray zone (5.5–6.4%). Results: HbA1c showed a strong continuous association with fasting plasma glucose (ρ = 0.73, *p* < 0.001) and was positively associated with cumulative complication burden (β = 0.016 per 1% increase in HbA1c, *p* = 0.009). Non-linear modeling revealed a progressive increase in complication risk beginning below the diagnostic threshold for diabetes, with an inflection of the risk curve within the HbA1c gray zone. Individuals within this interval exhibited a higher prevalence and increased odds of presenting at least one complication compared with lower HbA1c values, although some estimates did not reach statistical significance. Conclusions: HbA1c acts as a continuous and non-linear marker of metabolic stress, with potentially biologically meaningful increases in complication risk emerging below traditional diagnostic thresholds. We demonstrate a non-linear acceleration of microvascular risk within the 5.5–6.4% interval, rather than a simple linear gradient. These findings support the concept of a glycemic risk continuum and highlight the clinical relevance of the HbA1c sub-diagnostic interval for early risk stratification and preventive strategies.

## 1. Introduction

Glycated hemoglobin (HbA1c) is a central biomarker in the assessment of glycemic control and the diagnosis of diabetes mellitus. By reflecting average blood glucose levels over prolonged periods, HbA1c integrates both fasting and postprandial glycemic exposure. Despite its widespread clinical use, HbA1c is most often interpreted using fixed diagnostic thresholds, which may not fully capture the biological complexity of metabolic dysregulation [1,2,3].

Increasing evidence suggests that metabolic risk does not emerge abruptly at conventional diagnostic cut-offs but instead develops progressively across a continuum of glycemic values. Subclinical inflammation, endothelial dysfunction, oxidative stress, and glycemic variability have been proposed as mechanisms linking modest elevations in HbA1c to early tissue damage. These processes may contribute to the development of microvascular complications even before overt diabetes is diagnosed [4,5].

Prediabetes, defined by intermediate HbA1c values, is frequently regarded as a transitional stage. However, this categorization may obscure important heterogeneity within the intermediate glycemic range. Individuals with similar HbA1c values may differ substantially in metabolic stress, complication susceptibility, and clinical trajectories. Consequently, reliance on categorical classification alone may underestimate early risk and delay preventive interventions [6].

From a methodological perspective, analyzing HbA1c as a continuous variable offers several advantages. Continuous modeling preserves information lost through categorization and allows the identification of linear and non-linear risk patterns. In particular, non-linear approaches such as spline modeling can reveal inflection points and acceleration zones within the glycemic spectrum, providing insight into biologically meaningful thresholds that may not coincide with clinical definitions [7].

The concept of an HbA1c “gray zone,” typically spanning values between approximately 5.5% and 6.4%, has gained increasing attention as a potential window of early metabolic vulnerability. Clarifying whether this interval is associated with measurable increases in complication burden and risk is of considerable clinical relevance [8,9,10].

Therefore, the aim of the present study was to evaluate HbA1c as a continuous and non-linear marker of metabolic stress in relation to metabolic parameters and microvascular complication burden in a real-world cohort. While HbA1c is increasingly recognized as a continuous risk marker, few studies have translated this non-linear relationship into individualized risk estimation. The novelty of our study lies in combining non-linear modeling with cumulative microvascular burden to derive a clinically usable HbA1c-based risk framework.

## 2. Materials and Methods

### 2.1. Study Design and Population

This cross-sectional observational study included adult subjects with available data on HbA1c and metabolic parameters. Clinical and laboratory information was retrospectively extracted from an institutional database. Only subjects with complete HbA1c measurements and documented diabetes-related complication data were included in the analysis.

The study population represents a real-world clinical cohort with a wide range of glycemic values, allowing the evaluation of HbA1c as a continuous metabolic marker.

Instead of single-complication outcomes, we evaluated cumulative microvascular burden as a clinically relevant integrative endpoint.

### 2.2. Clinical and Metabolic Assessment

Demographic variables included age and sex. Glycemic status was assessed using HbA1c (%) and fasting plasma glucose (mg/dL), measured according to standard laboratory procedures.

HbA1c was analyzed exclusively as a continuous variable in all primary analyses. No categorical stratification based on diagnostic thresholds was applied, except for exploratory analyses focused on the HbA1c gray zone.

Common cardiometabolic comorbidities, including hypertension, dyslipidemia, and obesity, were recorded based on documented clinical diagnoses. Obesity was classified according to standard body mass index (BMI) categories.

Due to differences in database storage formats, HbA1c values were harmonized before analysis. Some entries were stored as integer values representing tenths of a percent (e.g., 81 = 8.1%). Values exceeding physiologically plausible percentages (>20) were interpreted as tenths and divided by 10. All HbA1c values were subsequently expressed uniformly in % units.

### 2.3. Assessment of Diabetes-Related Complications

Diabetes-related complications were identified from clinical records and included:

- peripheral neuropathy;
- diabetic retinopathy;
- diabetic nephropathy;
- peripheral arteriopathy.

In addition to individual complications, a cumulative complication burden was calculated for each subject as the total number of documented diabetes-related complications. For risk estimation analyses, a binary outcome variable indicating the presence of at least one complication was defined.

### 2.4. Development and Internal Validation of the HbA1c-Based Microvascular Risk Algorithm

To translate the observed HbA1c–microvascular associations into a clinically applicable framework, we developed an HbA1c-based risk algorithm for estimating the probability of presenting at least one diabetes-related microvascular complication.

Model Development

The primary outcome for the risk algorithm was the presence of ≥1 documented microvascular complication (binary variable), defined as peripheral neuropathy, diabetic retinopathy, diabetic nephropathy, or peripheral arteriopathy, as previously described.

Candidate predictors were selected a priori based on clinical relevance and data availability and included:

HbA1c (%)

- age (years)
- sex (male/female)
- hypertension (yes/no)
- dyslipidemia (yes/no)
- obesity (BMI-defined, yes/no)

HbA1c was modeled as a continuous variable using restricted cubic splines to account for potential non-linear associations with complication risk. Restricted cubic splines with three knots placed at standard percentiles.

A multivariable logistic regression model was constructed to estimate individualized probabilities of ≥1 complication. Regression coefficients from the final model were used to derive predicted risks.

#### 2.4.1. Cumulative Microvascular Load Modeling Methods

As a secondary analysis, the cumulative number of microvascular complications (0–4) was treated as a count outcome reflecting overall microvascular burden. Because count data may exhibit overdispersion, negative binomial regression models were applied. HbA1c was again modeled as a continuous predictor with non-linear terms. Incidence rate ratios (IRRs) and 95% confidence intervals (CIs) were reported.

#### 2.4.2. Internal Validation

Internal validation focused on discrimination (AUC), calibration plots, and Brier score. Bootstrap optimism correction was not performed, and model performance is, therefore, reported as apparent performance.

#### 2.4.3. Risk Presentation

To enhance clinical interpretability, predicted probabilities were presented as risk estimates across representative HbA1c values and age categories. These estimates were used to construct a practical HbA1c-based risk framework for early risk stratification.

### 2.5. Statistical Analysis

Descriptive statistics are presented as mean ± standard deviation, range, or number (percentage), as appropriate. Continuous associations between HbA1c and metabolic parameters were evaluated using Spearman correlation analysis.

Linear regression models were initially applied to descriptively assess the relationship between HbA1c (continuous predictor) and cumulative complication burden. The regression coefficient (β) represents the estimated change in the number of complications per 1% increase in HbA1c.

Because cumulative complication burden represents count data, negative binomial regression models were subsequently used as the primary inferential approach to account for overdispersion and to provide more robust estimates of the association between HbA1c and microvascular burden.

To explore non-linear associations between HbA1c and complication risk, restricted cubic spline logistic regression models were constructed, with HbA1c as a continuous predictor and the presence of at least one complication as the outcome. Spline analyses were restricted to biologically plausible HbA1c ranges to minimize distortion from extreme values. Restricted cubic spline models were constructed using three knots placed at standard percentiles of the HbA1c distribution, with the median HbA1c value used as the reference.

For gray-zone analyses, the HbA1c interval of 5.5–6.4% was compared to lower HbA1c values (<5.5%) using univariate logistic regression to estimate odds ratios (ORs) and 95% confidence intervals (CIs).

All statistical analyses were performed using IBM SPSS Statistics (version 30, IBM Corp., Armonk, NY, USA) and complementary regression modeling software. A two-sided *p*-value < 0.05 was considered statistically significant.

### 2.6. Ethical Considerations

The study was conducted in accordance with the Declaration of Helsinki and approved by the Institutional Ethics Committee of the Hospital (protocol No. 46/date 31 October 2025) and the Institutional Review Board (protocol code CEFMF/1 from 31 January 2023 and date of approval). Informed consent was obtained from all subjects involved in the study. Written informed consent has been obtained from the patients to publish this paper.

## 3. Results

### 3.1. Continuous Associations Between HbA1c and Metabolic Parameters

Study population characteristics

Table 1 presents the baseline demographic and metabolic characteristics of the 839 participants included in the analysis under a continuous HbA1c framework. The cohort had a mean age of 63.1 ± 10.2 years (range 27–90), indicating a broad adult age distribution with predominance in older age groups. Sex distribution was approximately balanced (48.5% male, 51.5% female), minimizing major sex imbalance as a potential confounder at the descriptive level.

HbA1c values demonstrated substantial dispersion (mean 7.63 ± 1.78%, range 4.6–18.0%), supporting the analytical treatment of HbA1c as a continuous exposure variable across a wide glycemic spectrum. Fasting plasma glucose levels were likewise variable (156.8 ± 59.0 mg/dL), consistent with heterogeneous glycemic control within the cohort.

Cardiometabolic comorbidities were highly prevalent, with hypertension and dyslipidemia each affecting approximately two-thirds of participants (66.4% and 66.3%, respectively), reflecting a population with elevated baseline cardiometabolic risk. Microvascular involvement was evidenced by documented nephropathies (24.0%) and retinopathies (14.8%), indicating a non-trivial burden of diabetes-related end-organ damage.

Anthropometric categorization showed that excess body weight predominated, with 77.1% of participants classified as overweight or obese. Obesity class I represented the largest subgroup (32.2%), followed by class II (14.8%) and class III obesity (6.6%), suggesting a right-shifted BMI distribution compatible with increased metabolic vulnerability.

The distribution of cumulative complications was right-skewed: 40.0% had no documented complications, whereas 60.0% presented ≥1 complication. Most affected individuals had a single complication (43.4%), while progressively fewer participants exhibited multimorbidity (≥2 complications: 16.5%). This gradient supports the suitability of the dataset for modeling complication burden as a count outcome and for exploring dose–response relationships with continuous HbA1c.

Overall, the observed variability in glycemic indices, comorbidity prevalence, and complication burden provides sufficient heterogeneity to support continuous and non-linear modeling strategies.

In contrast, no significant association was identified between HbA1c and age (ρ = −0.035, *p* = 0.304; n = 839) (Supplementary Figure S1).

### 3.2. HbA1c and Cumulative Complication Burden

Using HbA1c as a continuous variable, a significant positive association was observed between HbA1c levels and the cumulative number of diabetes-related complications. Linear regression analysis demonstrated that increasing HbA1c values were associated with a gradual increase in complication burden (β = 0.016 complications per 1% increase in HbA1c, *p* = 0.009) (Table 2).

Although the strength of the correlation was modest (r = 0.09), the association remained statistically significant across the full HbA1c spectrum, indicating that even incremental increases in HbA1c are associated with a higher cumulative burden of complications.

Negative binomial models yielded consistent directional results and were used to confirm the robustness of the association.

These findings support the concept of HbA1c as a continuous marker of metabolic stress, with progressive biological association rather than a strict threshold-dependent effect (Supplementary Figure S2).

### 3.3. Non-Linear Risk Patterns and the “Gray Zone”

To explore potential non-linear associations between HbA1c and complication risk, a restricted cubic spline logistic regression model was applied, using HbA1c as a continuous predictor and the presence of at least one diabetes-related complication as the outcome.

The spline analysis revealed a non-linear relationship between HbA1c levels and complication risk. A gradual increase in the predicted probability of presenting at least one complication was observed starting at HbA1c values below the conventional diagnostic threshold for diabetes. Notably, the slope of the risk curve increased within the HbA1c range of approximately 5.5–6.4%, corresponding to the clinically defined prediabetic interval.

Extremely high HbA1c values likely reflect advanced disease states and were not interpreted individually; their influence on non-linear models was mitigated by range restriction.

Beyond this range, the probability of complications continued to rise with increasing HbA1c values, with a more pronounced acceleration at higher levels. These findings indicate that complication risk does not emerge abruptly at diagnostic cut-offs but follows a continuous and non-linear biological gradient (Supplementary Figure S3).

### 3.4. Risk Estimation Within the HbA1c Gray Zone (5.5–6.4%)

To further quantify the biological relevance of the HbA1c gray zone, a risk estimation analysis was performed by comparing individuals with HbA1c values between 5.5% and 6.4% to those with HbA1c levels below 5.5%. The presence of at least one diabetes-related complication was used as the outcome.

Subjects within the intermediate HbA1c range exhibited a higher prevalence of at least one complication compared to the reference group (56.3% vs. 44.4%). Logistic regression analysis indicated an increased odds of presenting at least one complication among intermediate glycemic spectrum individuals (OR = 1.61, 95% CI: 0.73–3.59); however, this association did not reach statistical significance (*p* = 0.240), likely due to the limited size of the reference subgroup with HbA1c < 5.5%.

In an additional continuous analysis restricted to the HbA1c interval of 5.0–6.4%, each 0.1% increase in HbA1c was associated with an estimated 5% increase in the odds of presenting at least one complication (OR per 0.1% = 1.05, 95% CI: 0.98–1.13; *p* = 0.193). Although not statistically significant, this trend is consistent with the non-linear risk pattern observed in the spline analysis.

Although the analyses suggested a higher prevalence and a directional increase in complication risk within the HbA1c gray zone, these estimates did not reach statistical significance and should therefore be interpreted cautiously. The observed patterns should be considered exploratory signals consistent with the spline-derived risk trajectory rather than definitive evidence of increased risk (Supplementary Figure S4).

### 3.5. Development and Performance of the HbA1c-Based Microvascular Risk Algorithm

#### 3.5.1. Multivariable Model for ≥1 Microvascular Complication

A multivariable logistic regression model was developed to estimate the probability of presenting at least one diabetes-related microvascular complication.

In the adjusted model, HbA1c demonstrated a significant non-linear association with complication risk when modeled using restricted cubic splines. The overall contribution of the HbA1c spline term to the model was statistically significant (overall *p* for HbA1c < 0.01), supporting a non-linear HbA1c–risk relationship.

Among covariates, older age, hypertension, and obesity were independently associated with a higher probability of microvascular complications, whereas sex and dyslipidemia showed weaker associations.

**Figure 1:**
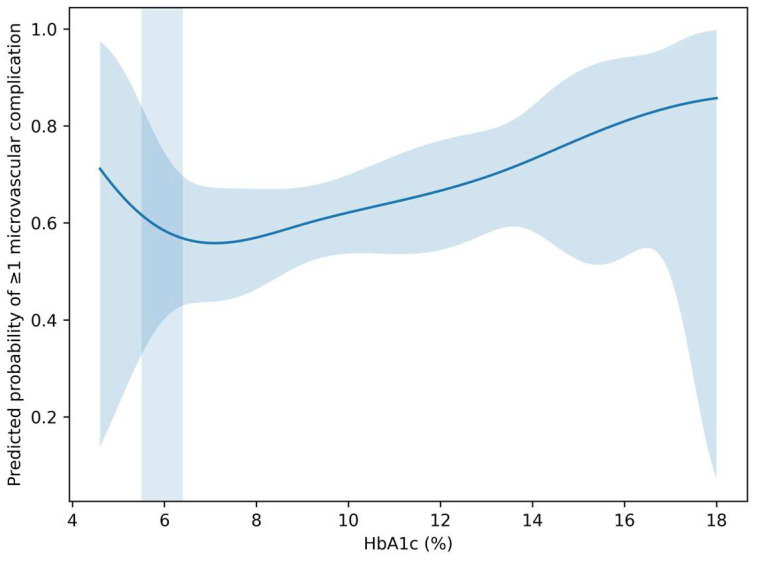
Adjusted non-linear association between HbA1c and microvascular complication risk. Predicted probability of presenting ≥1 microvascular complication across the HbA1c continuum, estimated using a multivariable logistic regression model with restricted cubic splines for HbA1c. The solid line represents the adjusted predicted probability, and the shaded area indicates the 95% confidence interval. The highlighted vertical band denotes the HbA1c gray zone (5.5–6.4%). Predictions are adjusted for age, sex, hypertension, dyslipidemia, and obesity (covariates held at their reference/typical values as defined by the model).

HbA1c showed a non-linear association with the probability of ≥1 microvascular complication in the adjusted spline model (Figure 1).

Importantly, the predicted probability of ≥1 complication increased progressively across the HbA1c continuum, with a visible risk acceleration within the 5.5–6.4% interval. This pattern persisted after multivariable adjustment, indicating that the gray-zone effect was not solely attributable to major cardiometabolic comorbidities.

#### 3.5.2. Model Discrimination and Calibration

The HbA1c-based risk model demonstrated modest but acceptable discriminative ability, with an apparent AUC of 0.64, indicating fair separation between individuals with and without microvascular complications.

Calibration analysis showed a generally good agreement between predicted and observed risks. Visual inspection of the calibration plot suggested no major systematic over- or underestimation across most risk ranges.

The Brier score was 0.225, consistent with acceptable overall model performance for a clinical prediction model in a heterogeneous real-world cohort.

**Figure 2:**
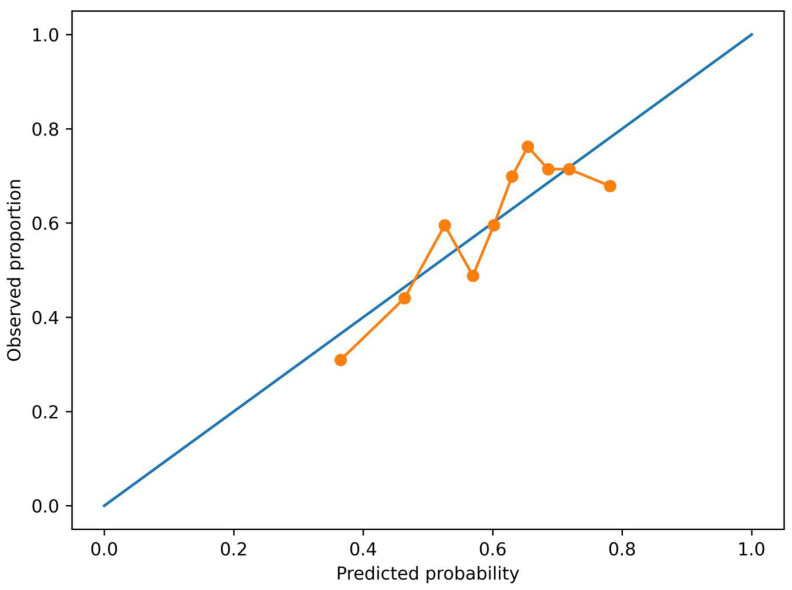
Calibration of the HbA1c-based microvascular risk model. Calibration plot comparing predicted and observed probabilities of ≥1 microvascular complication derived from the multivariable logistic regression model. The 45° diagonal line indicates perfect calibration. The calibration curve illustrates model performance across increasing levels of predicted risk. Close agreement between the two lines suggests satisfactory calibration and limited systematic risk over- or underestimation. The blue diagonal line represents perfect calibration (ideal predictions). The orange line with markers represents the observed outcome frequencies across grouped predicted-probability intervals.

A calibration plot across deciles of predicted risk demonstrated that the model provided reasonably stable risk estimates in the low- and intermediate-risk ranges, which are particularly relevant for early preventive strategies (Figure 2).

#### 3.5.3. Risk Estimates Across the HbA1c Spectrum

To facilitate clinical interpretation, predicted probabilities were examined across representative HbA1c levels.

At lower HbA1c values (e.g., ~5.0–5.4%), the estimated probability of ≥1 complication remained relatively low. However, beginning around HbA1c values of approximately 5.5–6.0%, the slope of the risk curve increased, indicating a transition from low to intermediate risk.

Within the 5.5–6.4% interval, small incremental increases in HbA1c were associated with disproportionately larger increases in predicted risk compared to lower HbA1c ranges. This supports the concept of a risk acceleration zone rather than a simple linear gradient.

**Figure 3:**
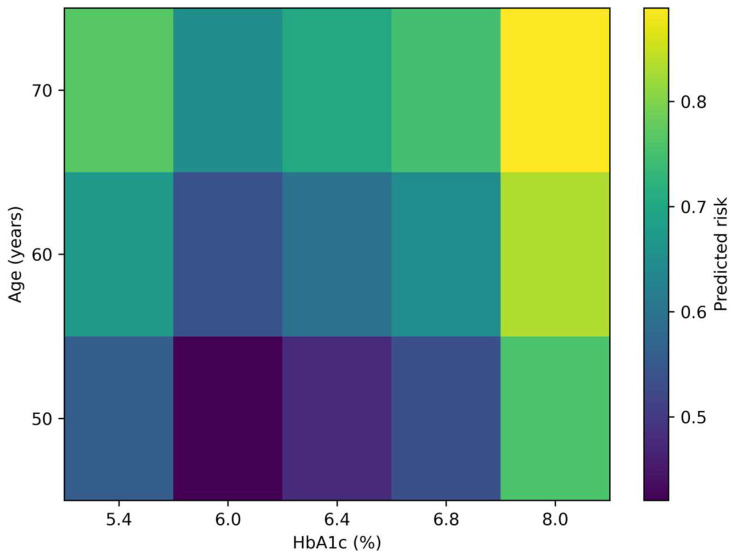
Clinical risk heatmap derived from the HbA1c-based microvascular risk model. Heatmap illustrating predicted probabilities of presenting ≥1 microvascular complication across representative HbA1c values and age categories. Color gradients reflect increasing predicted risk from lower (purple/blue) to higher (yellow) probabilities. Estimates are derived from the multivariable model and adjusted for sex, hypertension, dyslipidemia, and obesity (held at typical/reference values).

At higher HbA1c levels (>7%), the predicted probability of complications rose more steeply, consistent with advanced metabolic stress and cumulative glycemic exposure (Figure 3).

#### 3.5.4. Modeling of Cumulative Microvascular Burden

In negative binomial regression analyses, higher HbA1c values were associated with a greater number of microvascular complications.

Each 1% increase in HbA1c was associated with an increased microvascular burden (*p* < 0.05). When modeled non-linearly, the HbA1c–burden relationship showed a similar pattern to the binary outcome, with early upward deviation of risk in the intermediate HbA1c range.

**Figure 4:**
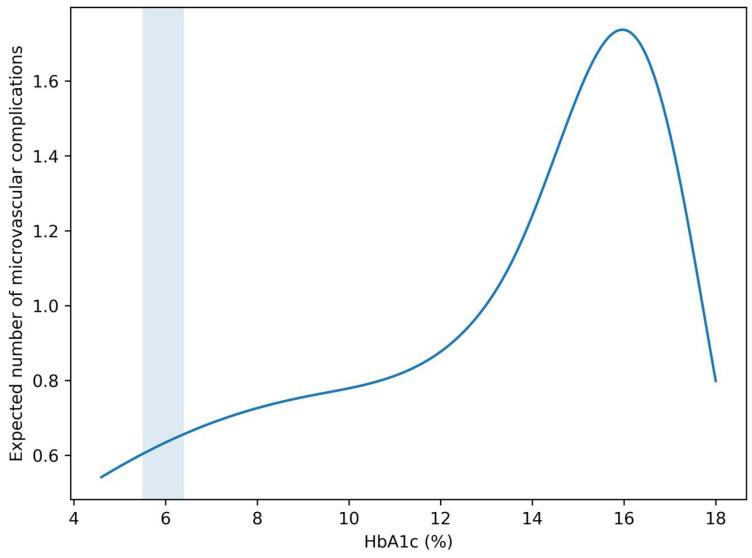
Association between HbA1c and cumulative microvascular burden. Predicted number of diabetes-related microvascular complications across the HbA1c continuum, derived from a negative binomial regression model. The curve represents the expected number of complications as a function of HbA1c, adjusted for age, sex, hypertension, dyslipidemia, and obesity. The upward trend indicates increasing microvascular burden with higher HbA1c levels. The shaded area denotes the HbA1c range corresponding to prediabetes.

These findings indicate that HbA1c is associated not only with the presence but also with the accumulation of microvascular complications (Figure 4).

#### 3.5.5. Clinical Translation

Based on these results, we derived an HbA1c-based risk framework that allows estimation of individualized microvascular risk along the glycemic continuum. This framework is particularly informative in individuals with HbA1c values below diagnostic thresholds, where traditional categorical approaches may underestimate risk.

## 4. Discussion

In this study, we investigated HbA1c as a continuous marker of metabolic stress, exploring both linear and non-linear associations with metabolic parameters and diabetes-related complication burden. By moving beyond conventional diagnostic thresholds, our findings provide evidence consistent with a progressive and non-linear risk continuum, with biologically meaningful changes occurring within the intermediate HbA1c range.

### 4.1. Continuous HbA1c Variation and Metabolic Stress

Our results suggest a strong continuous association between HbA1c and fasting plasma glucose, supporting the validity of HbA1c as an integrated marker of chronic glycemic exposure across a wide metabolic spectrum. In contrast, HbA1c showed no significant correlation with age, suggesting that the observed associations are primarily driven by metabolic rather than demographic factors.

Importantly, when analyzed continuously, HbA1c was significantly associated with the cumulative number of diabetes-related complications, even though the effect size per unit increase was modest. This finding reflects the multifactorial nature of complication development and underscores the relevance of small but cumulative increases in glycemic burden over time [11,12,13,14,15,16].

The observed effect sizes were modest, reflecting the multifactorial nature of complication burden. While statistically significant, these associations should not be interpreted as clinically deterministic at the individual level. Large cohort studies have reported graded HbA1c–outcome relationships even below diabetic thresholds. Our findings extend this literature by characterizing the shape of this association using non-linear modeling in a real-world cohort.

### 4.2. Non-Linear Risk Patterns and the Biological Relevance of the Gray Zone

A key contribution of this study is the identification of non-linear risk patterns linking HbA1c to complication susceptibility. Restricted cubic spline analyses revealed that the probability of presenting at least one complication begins to increase at HbA1c levels below the diagnostic threshold for diabetes, with a noticeable inflection within the 5.5–6.4% interval.

Risk estimation analyses within this sub-diagnostic interval further supported its biological relevance, showing higher complication prevalence and increased odds compared to lower HbA1c values. Although some associations did not reach statistical significance, likely due to limited subgroup size, the consistent direction of effects aligns with the spline-derived risk trajectory [17,18,19,20,21].

These findings challenge the biological rigidity of diagnostic cut-offs and support the view that HbA1c thresholds represent clinical conventions rather than biological boundaries. The gray zone may, therefore, represent a window of opportunity for early intervention before overt diabetes develops [9,22,23,24,25].

Several large cohort and meta-analytic studies have demonstrated graded relationships between HbA1c and vascular outcomes, even within non-diabetic ranges. Our findings extend this literature by showing that similar gradients are detectable at the level of cumulative microvascular burden in a real-world cohort and by characterizing the shape of this relationship using non-linear modeling. This supports the concept that HbA1c-related risk is not only continuous but also potentially non-linear in its biological expression.

#### Clinical Relevance of Optimal HbA1c Beyond Diabetes Diagnosis

While HbA1c is primarily used to diagnose and monitor diabetes, growing evidence suggests that its clinical relevance extends beyond overt hyperglycemia. HbA1c values within the so-called “gray zone” may reflect early metabolic stress, endothelial dysfunction, and low-grade inflammation even in individuals without diagnosed diabetes. In this context, modest elevations in HbA1c have been associated with adverse renal and neurological outcomes, including impaired nephroprotection in patients with renovascular hypertension undergoing renal artery interventions and increased long-term risk of cognitive decline and dementia. These observations support the concept that optimal glycemic exposure should be considered along a biological continuum rather than defined solely by diagnostic thresholds. Our findings reinforce this view by demonstrating a progressive increase in complication susceptibility beginning below the conventional diabetes cut-off, highlighting the potential value of HbA1c as an early, integrative risk marker in both diabetic and non-diabetic populations [26,27].

### 4.3. Clinical and Methodological Implications

From a clinical perspective, our results suggest that reliance solely on categorical HbA1c thresholds may underestimate early metabolic and microvascular risk. Continuous HbA1c assessment and risk modeling may provide a more nuanced framework for patient stratification, particularly in individuals with intermediate glycemic values.

Methodologically, this study highlights the added value of non-linear modeling approaches, such as spline analyses, in uncovering risk patterns that are not apparent in categorical or purely linear analyses. Such approaches may enhance risk prediction and support personalized preventive strategies [28,29,30,31].

From a translational perspective, these findings suggest that individuals in the HbA1c gray zone may benefit from earlier risk-oriented evaluation rather than simple categorical labeling as prediabetic. Continuous HbA1c-based risk assessment could help identify patients who might otherwise be overlooked under threshold-based frameworks, particularly in settings focused on complication prevention.

Beyond microvascular outcomes, intermediate glycemic dysregulation has also been linked to subclinical myocardial dysfunction, including alterations in the myocardial performance index, supporting broader cardiometabolic vulnerability across the glycemic continuum [32].

Taken together, the four complementary visualizations presented in this study provide a coherent picture of HbA1c as a continuous and clinically actionable marker of microvascular risk. The adjusted spline curve (Figure 1) demonstrates that the HbA1c–risk relationship is not simply linear but shows gradual risk acceleration, particularly within the sub-diagnostic range. The calibration analysis (Figure 2) indicates that this relationship can be translated into reasonably reliable individual-level risk estimates in a real-world cohort. Moving beyond model validity, the risk heatmap (Figure 3) illustrates how the continuous HbA1c–risk gradient can be operationalized into a clinically interpretable framework, allowing visualization of how small HbA1c differences and age interact to modify predicted risk. Finally, the burden analysis (Figure 4) extends these findings by showing that HbA1c is associated not only with the presence of complications but also with their cumulative load. Considered together, these findings shift the perspective from HbA1c as a threshold-based diagnostic marker to HbA1c as a quantitative indicator of progressive microvascular vulnerability, supporting a continuum-based approach to early risk stratification.

### 4.4. Strengths and Limitations

First, the cross-sectional design of the study precludes any inference regarding causal relationships or temporal ordering between HbA1c levels and the development of microvascular complications. Consequently, the observed associations should be interpreted as descriptive patterns within a real-world cohort rather than evidence of causality. The findings primarily generate hypotheses regarding potential early metabolic vulnerability associated with intermediate HbA1c levels, which should be further investigated in prospective longitudinal studies.

Second, several potentially relevant confounding variables were not available in the institutional database and, therefore, could not be included in the multivariable models. These include diabetes duration, antidiabetic treatment regimens, smoking status, renal function parameters, and systemic inflammatory markers. Because these factors are known to influence both glycemic control and the development of microvascular complications, their absence may have affected the magnitude and precision of the observed associations. Future studies incorporating a broader range of clinical and biochemical variables would allow more comprehensive adjustment for confounding and improve the interpretability of risk estimates.

Third, although model discrimination and calibration were evaluated, formal internal validation procedures such as bootstrap optimism correction or cross-validation were not performed. As a result, the reported performance metrics represent apparent model performance and may be subject to optimism bias. External validation in independent cohorts will be necessary before the proposed HbA1c-based risk framework can be considered for broader clinical application.

Given the cross-sectional design, all observed associations should be interpreted as descriptive and hypothesis-generating, without inference on temporal sequence or causality.

Important confounders, including diabetes duration, treatment modality, renal function, smoking status, and inflammatory markers, were not available for inclusion in multivariable models. Their omission may have influenced the magnitude and precision of observed associations.

### 4.5. Future Directions

Overall, this study is consistent with the concept of HbA1c as a continuous and non-linear marker of metabolic stress, with clinically relevant risk emerging below conventional diagnostic thresholds. Future longitudinal studies are needed to confirm these findings and to determine whether early intervention in the HbA1c gray zone can effectively reduce long-term complication risk.

## 5. Conclusions

This study provides descriptive evidence suggesting that HbA1c may function as a continuous and non-linear marker of metabolic stress rather than a purely threshold-dependent diagnostic parameter. Across a wide HbA1c spectrum, increasing values were associated with progressive changes in metabolic status, cumulative complication burden, and estimated complication risk.

Importantly, biologically meaningful increases in complication susceptibility were observed within the HbA1c gray zone (5.5–6.4%), supporting the concept that metabolic risk emerges gradually and precedes the conventional diagnostic threshold for diabetes. Non-linear modeling further revealed that risk acceleration does not occur abruptly at diagnostic cut-offs but follows a continuous trajectory.

These findings should be interpreted cautiously and primarily as hypothesis-generating, and they highlight the potential clinical and methodological value of treating HbA1c as a continuous variable for risk stratification and early identification of vulnerable individuals. Incorporating continuous and non-linear HbA1c-based risk assessment into clinical practice may help inform preventive strategies and support more personalized approaches to metabolic health.

## Figures and Tables

**Table 1:** Demographic and metabolic characteristics of the study population (continuous HbA1c approach).

Variable	Value
Number of participants	839
Age (years), mean ± SD	63.1 ± 10.2
Age range (years)	27–90
Sex, *n* (%)	
Male	407 (48.5%)
Female	432 (51.5%)
HbA1c (%), mean ± SD	7.63 ± 1.78
HbA1c range (%)	4.6–18.0
Fasting glucose (mg/dL), mean ± SD	156.8 ± 59.0
HTN, *n* (%)	557 (66.4%)
Dyslipidemia	556 (66.3%)
Nephropathies	201 (24.0%)
Retinopathies	124 (14.8%)
Weight status	
Normal	192 (22.9%)
Overweight	198 (23.6%)
Obesity degree I	270 (32.2%)
Obesity degree II	124 (14.8%)
Obesity degree III	55 (6.6%)
Number of complications	
0	336 (40.0%)
1	364 (43.4%)
2	110 (13.1%)
3	28 (3.3%)
4	1 (0.1%)

Using HbA1c as a continuous variable, we observed a strong positive monotonic association between HbA1c and fasting plasma glucose. Spearman correlation analysis showed a strong correlation between HbA1c and fasting glucose (ρ = 0.728, *p* < 0.001; *n* = 839), supporting a consistent glycemic gradient across the full HbA1c spectrum (HTN: hypertension).

**Table 2:** Linear association between HbA1c and cumulative complication burden.

Predictor	Outcome	β (Slope)	Standard Error	r	*p*-Value
HbA1c (%)	Number of complications	0.016	0.006	0.09	0.009

Linear regression analysis was performed with the cumulative number of diabetes-related complications as the dependent variable and HbA1c (%) as a continuous predictor. β represents the estimated change in the number of complications per 1% increase in HbA1c.

## Data Availability

The original contributions presented in this study are included in the article/Supplementary Materials. Further inquiries can be directed to the corresponding author(s).

## Supplementary Materials

The following supporting information can be downloaded at: https://www.mdpi.com/article/10.3390/metabo16030197/s1, Figure S1: HbA1c and fasting glucose: continuous association (scatter plot). Figure S2: Association between HbA1c and cumulative complication burden. Figure S3: Non-linear association between HbA1c and complication risk. Figure S4: Complication risk in the HbA1c gray zone.

## Abbreviations

The following abbreviations are used in this manuscript:

BMI	Body Mass Index
CI	Confidence Interval
HbA1c	Glycated Hemoglobin
HTN	Hypertension
OR	Odds Ratio
SD	Standard Deviation
