# Supplementary material for: HbA1c as a Continuous Marker of Microvascular Vulnerability: Development of a Non-Linear Risk Framework in a Real-World Cohort
Source: Metabolites. 2026 Mar 16;16(3):197. doi: 10.3390/metabo16030197 (PMC13029085; doi:10.3390/metabo16030197)
Supplement: Supplementary file 1 [file metabolites-16-00197-s001.zip › metabolites-4177849-supplementary.pdf]

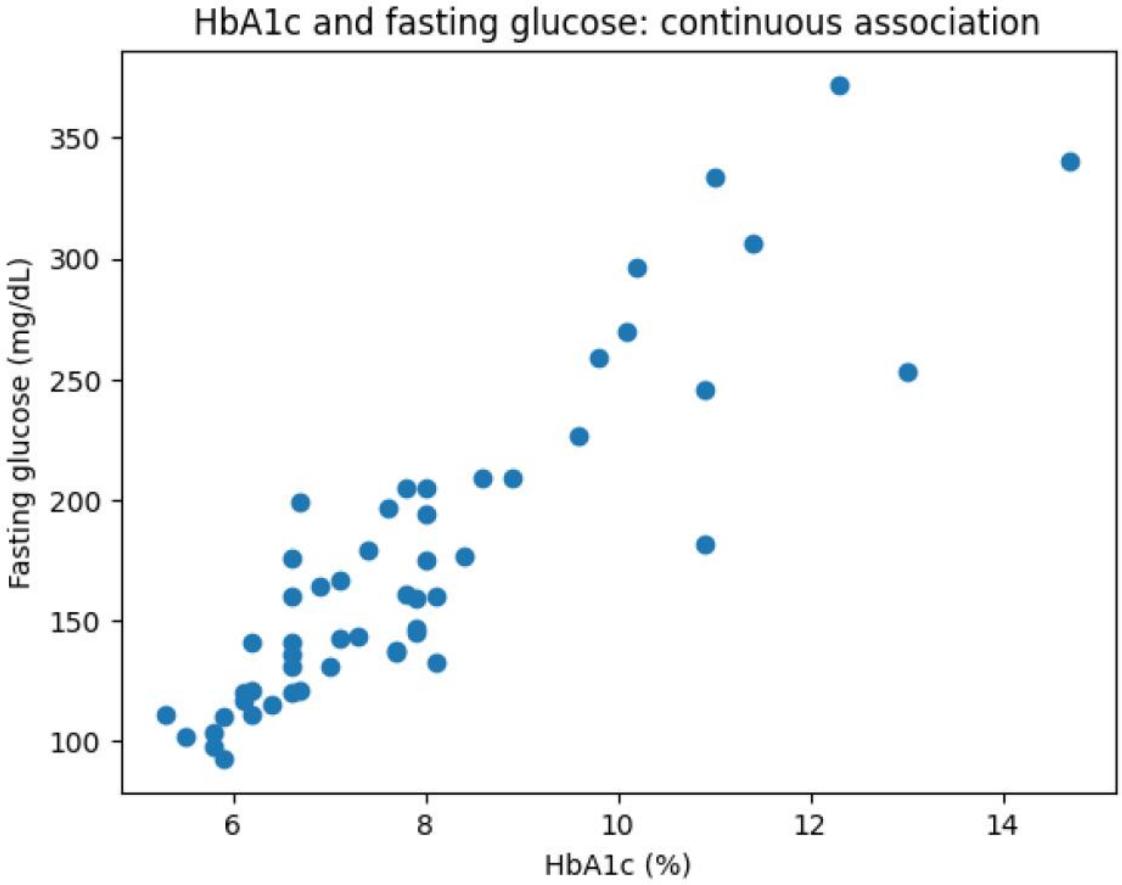

Figure S1. HbA1c and fasting glucose: continuous association.

Scatter plot illustrating the continuous relationship between glycated hemoglobin (HbA1c, %) and fasting plasma glucose (mg/dL). Each point represents an individual participant.

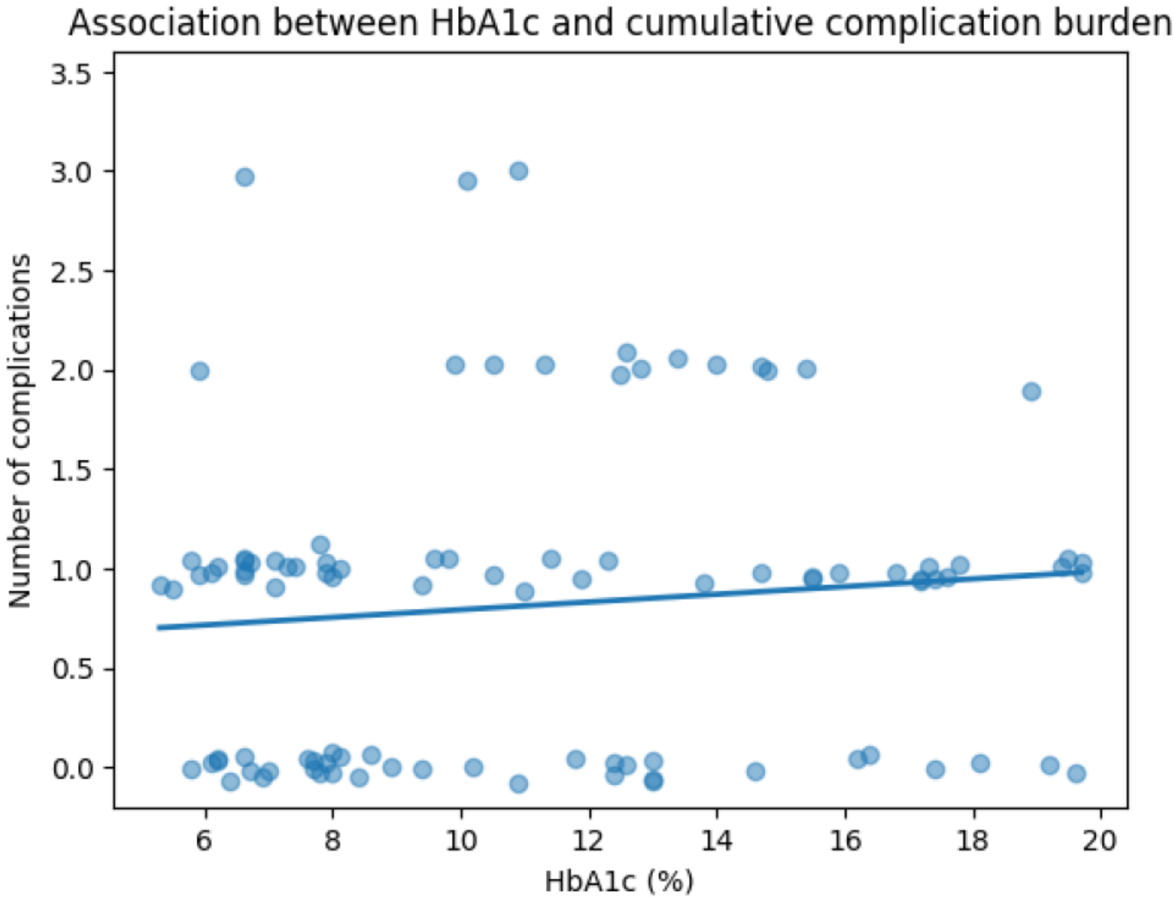

Figure S2. Association between HbA1c and cumulative complication burden.

Scatter plot showing the relationship between HbA1c (%) and the cumulative number of diabetes-related complications. The solid line represents the fitted linear regression model.

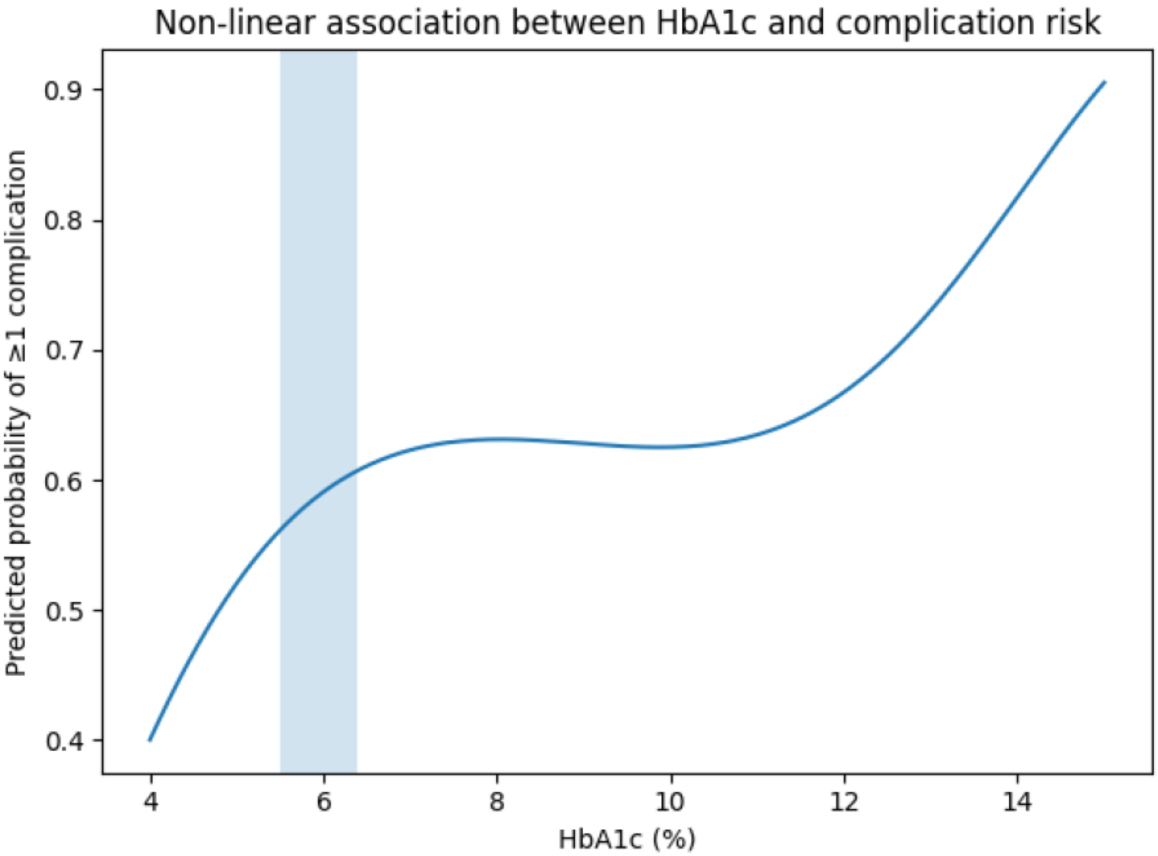

Figure S3. Non-linear association between HbA1c and complication risk.

Restricted cubic spline curve illustrating the non-linear relationship between HbA1c (%) and the predicted probability of presenting at least one diabetes-related complication. The shaded vertical band denotes the intermediate glycemic ("gray zone") range (HbA1c 5.5–6.4%), where an early increase in complication risk is observed.

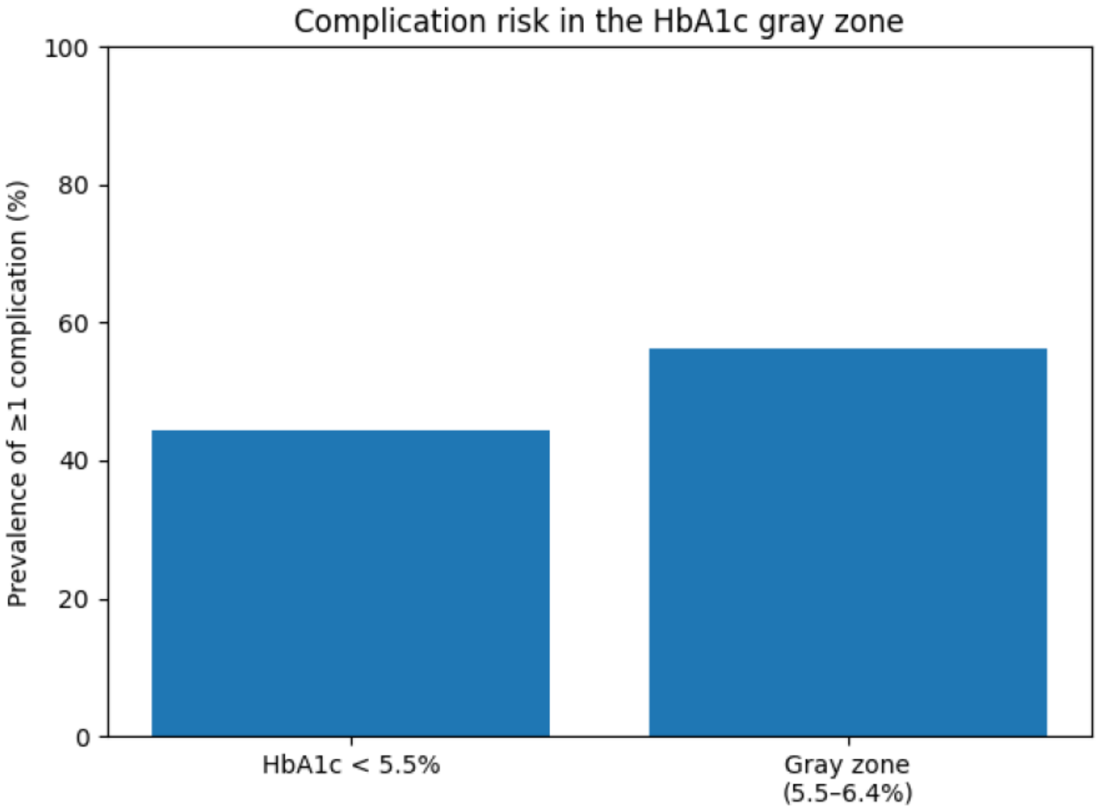

Figure S4. Complication risk within the HbA1c gray zone.

Bar chart showing the prevalence of at least one diabetes-related complication among individuals with HbA1c < 5.5% compared with those within the intermediate glycemic spectrum (HbA1c 5.5–6.4%). Individuals in the gray-zone range exhibit a higher prevalence of complications, supporting the concept of progressive metabolic risk below conventional diagnostic thresholds.
